# A Survey of Multigenic Protein-Altering Variant Frequency in Familial Exudative Vitreo-Retinopathy (FEVR) Patients by Targeted Sequencing of Seven FEVR-Linked Genes

**DOI:** 10.3390/genes13030495

**Published:** 2022-03-11

**Authors:** Amanda Petrelli Cicerone, Wendy Dailey, Michael Sun, Andrew Santos, Daeun Jeong, Lance Jones, Konstaninos Koustas, Mary Drekh, Keaton Schmitz, Naomi Haque, Jennifer A. Felisky, Alvaro E. Guzman, Kendra Mellert, Michael T. Trese, Antonio Capone, Kimberly A. Drenser, Kenneth P. Mitton

**Affiliations:** 1 Eye Research Institute, Rochester, MI 48309, USA; amandapetrellic@oakland.edu (A.P.C.); dailey@oakland.edu (W.D.); michaelsun@oakland.edu (M.S.); acsantos@oakland.edu (A.S.); djeong2@oakland.edu (D.J.); lancejones@oakland.edu (L.J.); kkoustas@oakland.edu (K.K.); mdrekh@oakland.edu (M.D.); kmschmitz@oakland.edu (K.S.); nmhaque@oakland.edu (N.H.); jafelisky@oakland.edu (J.A.F.); aeguzman@oakland.edu (A.E.G.); 2 Oakland University William Beaumont School of Medicine, Rochester, MI 48309, USA; 3 Associated Retinal Consultants LLC, Royal Oak, MI 48073, USA; kmellert@arcpc.net (K.M.); mtrese@arcpc.net (M.T.T.); acaponejr@arcpc.net (A.C.)

**Keywords:** FEVR, retinal disease, pediatric, inherited retinal disease, DNA sequencing, targeted sequencing, NGS, multigenic, protein variants

## Abstract

While Inherited Retinal Diseases (IRDs) are typically considered rare diseases, Familial Exudative Vitreo-Retinopathy (FEVR) and Norrie Disease (ND) are more rare than retinitis pigmentosa. We wanted to determine if multigenic protein-altering variants are common in FEVR subjects within a set of FEVR-related genes. The potential occurrence of protein-altering variants in two different genes has been documented in a very small percentage of patients, but potential multigenic contributions to FEVR remain unclear. Genes involved in these orphan pediatric retinal diseases are not universally included in available IRD targeted-sequencing panels, and cost is also a factor limiting multigenic-sequence-based testing for these rare conditions. To provide an accurate solution at lower cost, we developed a targeted-sequencing protocol that includes seven genes involved in Familial Exudative Vitreo-Retinopathy (FEVR) and Norrie disease. Seventy-six DNA samples from persons refered to clinic with possible FEVR and some close relatives were sequenced using a novel Oakland-ERI orphan pediatric retinal disease panel (version 2) providing 900 times average read coverage. The seven genes involved in FEVR/ND were: *NDP* (ChrX), *CTNNB1* (Chr3); *TSPAN12* (Chr7); *KIF11* (Chr10), *FZD4* (Chr11), *LRP5* (Chr11), *ZNF408* (Chr11). A total of 33 variants were found that alter protein sequence, with the following relative distribution: *LRP5* 13/33 (40%), *FZD4* 9/33 (27%), *ZNF408* 6/33 (18%), (*KIF11* 3/33 (9%), *NDP* 1/33 (3%), *CTNNB1* 1/33 (3%). Most protein-altering variants, 85%, were found in three genes: *FZD4*, *LRP5*, and *ZNF408*. Four previously known pathogenic variants were detected in five families and two unrelated individuals. Two novel, likely pathogenic variants were detected in one family (FZD4: Cys450ter), and a likely pathogenic frame shift termination variant was detected in one unrelated individual (LRP5: Ala919CysfsTer67). The average number of genes with protein-altering variants was greater in subjects with confirmed FEVR (1.46, *n* = 30) compared to subjects confirmed unaffected by FEVR (0.95, *n* = 20), (*p* = 0.009). Thirty-four percent of persons sequenced had digenic and trigenic protein-altering variants within this set of FEVR genes, which was much greater than expected in the general population (3.6%), as derived from GnomAD data. While the potential contributions to FEVR are not known for most of the variants in a multigenic context, the high multigenic frequency suggests that potential multigenic contributions to FEVR severity warrant future investigation. The targeted-sequencing format developed will support such exploration by reducing the testing cost to $250 (US) for seven genes and facilitating greater access to genetic testing for families with this very rare inherited retinal disease.

## 1. Introduction

FEVR (Familial Exudative Vitreo-retinopathy) and Norrie disease are inherited disorders that affect the development of the neural retinal vasculature, resulting in avascular regions in the peripheral retina [1,2,3]. FEVR was first described in 1968 and can result in blindness from retinal traction, folding, detachments, neovasuclarization, and vitreous hemorrhage [4]. FEVR phenotypically can show large variations in severity, even within a single family, and Shastry and Trese (2004) were the first to suggest that digenic alleles could exist in some FEVR families as a factor contributing to the variable penetrance [5]. More recently, Li et al. [6] reported evidence that digenic protein-altering variants occurred in 2.7% of their study cohort (13/487) using a four-gene panel and suggested that increasing the number of FEVR-linked genes sequenced might increase this percentage [6]. For this study, our goal was to increase the number of genes sequenced to seven genes. Currently, variants assocated with FEVR impact at least seven proteins (genes): Norrin Cystine-Knot Growth Factor NDP (*NDP),* Catenin β-1 *(CTNNB1),* Frizzled-4 (*FZD4*), Kinesin Family Member-11 (*KIF11*), LDL-Receptor-Related Protein-5 (*LRP5*), Tetraspanin-12 (*TSPAN12*), and Zinc-Finger Protein-408 (*ZNF408*). Due to the rarity of some of these conditions, DNA testing is still difficult to access, expensive, not available in most countries, and not supported by medical insurance in the United States.

We first pre-tested an amplicon targeted-sequencing panel with six FEVR-related genes and one for retinoschisis, and we confirmed that it detected known variants from 15 subjects that we had analyzed in years past using traditional PCR and Sanger sequencing. After that preliminary feasiblity test, we settled on using the Illumina AmpliSeq targeted-sequencing process to develop our current panel (version 2) for eight genes involved in three very rare pediatric retinal conditions: FEVR, Norrie disease, and retinoschisis. The seven FEVR/Norrie disease-related genes listed above are included. Using the AmpliSeq workflow for sequencing on the iSeq-100 instrument, the cost of testing was reduced to only $250 per sample ($32 US per gene), providing a practical research-sequencing service for families with these rare IRDs. The goal of this initial study was to ask if a significant percentage of FEVR subjects may have multigenic protein-altering variants in these seven FEVR-relevant genes. We then analyzed a novel group of 76 persons for DNA sequence variants that alter the amino acid sequence of the seven proteins of interest.

**Figure 1:**
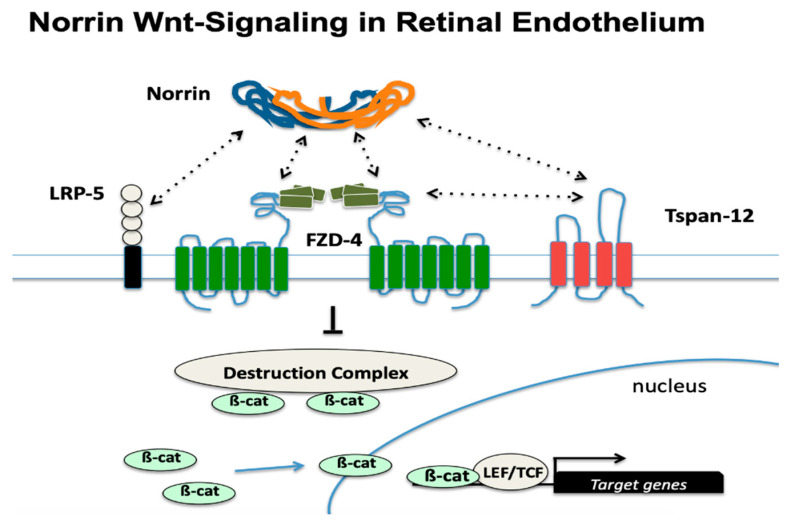
Norrin-based Wnt-signaling in retinal endothelial cells. Lack of Norrin-based Wnt-signaling results in underdevelopment of the microvascular beds and failure to develop a patent high-barrier vascular endothelium in the neural retina and in the cochlea. A Norrin dimer can bind to its central cognate receptor Frizzled-4 (FZD-4) as well as the co-receptors LRP-5 and TSPAN-12. Activation of the receptor complex is increased by the synergy of multiple protein–protein interactions (dashed arrows), inhibiting the ubiquitination of phosphorylated β-Catenin, which then accumulates on the destruction complex [11]. When Norrin is available, this effect causes an increase in cytoplasmic β-Catenin concentration, which enters the nucleus to modulate target gene expression through interactions with the TCF/LEF family of transcription factors as well as other transcription factors that remain unknown.

All the genes of interest are important to the biological function of retinal vascular endothelial cells. Most of the gene products are members of the Norrin-based signaling pathway specific to endothelial cells of the neural retinal microvasculature. Retinal endothelial cells are reliant on Norrin with their unique combination of receptor proteins that make them sensitive and responsive to Norrin. This combination consists of the co-expression of the FZD-4, TSPAN-12, and LRP-5 proteins (see Figure 1). While different Wnt family members can bind to FZD-4 in other cell types, the combination with LRP-5 and TSPAN-12 is required for the unique and strong response to Norrin [7]. In addition to the five proteins shown in Figure 1, two other proteins that are abundantly expressed and particularly required in retinal endothelial cells are KIF-11 and ZNF 408. ZNF 408 is a zinc-finger transcription factor that is essential for the adequate expression of genes required for normal endothelial cell function, and protein-altering variants of this gene are also reported to cause FEVR [8]. KIF-11 is the predominant Kinesin protein family member expressed in retinal endothelial cells. KIF-11 variants are also associated with several medical conditions including microcephaly, lymphedema, and chorioretinal dysplasia (MLCRD) as well as chorioretinal dysplasia, microcephaly, and mental retardation (CDMMR) [9,10]. Patients with KIF-11 mutations can have one or more of these conditions in combination.

Due to the rarity and complexity of these diseases, diagnosing FEVR can be very challenging. In addition, their polygenic nature makes identifying the cause difficult because of the small population of patients and the limited amount of research available [12]. Understanding the potential role of multiple protein-altering variants in more than one FEVR-relevant gene in the same patient faces two challenges. First, obtaining enough persons with these very rare conditions to consent for analysis is difficult. Second, an economical methodology is required to accurately sequence not just one, two, or four genes but a multigene panel that includes as many FEVR-related genes as possible. The first challenge was solved by the fact that the Associated Retinal Consultants practice (Royal Oak, MI, USA) is a tertiary referral center that sees FEVR patients from all over the United States and the world. The second challenge remaining to be solved was addressed here by the development of a custom AmpliSeq targeted-sequencing panel.

Before we can start to address the question of potential multigenic involvement in FEVR, we must first answer this question: what percentage of FEVR subjects have multiple protein-altering variants in this pool of seven FEVR-related genes? That question is the subject of this report. If multigenic protein-altering variants are not common in this pool of the most frequently involved FEVR-related genes, then it is unlikely we would need to be highly concerned with the potential for multigenic contributions in most FEVR subjects. On the other hand, if a significant proportion of FEVR subjects have multigenic protein-altering variants, then the multiple combinations would warrant some consideration in the analysis of families with this condition.

## 2. Materials and Methods

### 2.1. IRB Protocol and Test Group Characteristics

All of the human DNA sequencing work used for this research was accomplished under the approval of the Oakland University Institutional Research Committee through an IRB management agreement for the Creation of an Ophthalmology BioBank (OU-IRB approval #1314454-2) with the Western IRB (WIRB). Subjects providing DNA consented under WIRB-approved protocols for the DNA Eye-bank at Associated Retinal Consultants (ARC, Royal Oak, MI, USA) and for DNA-sequencing.

For this analysis, 76 persons’ samples were processed for targeted DNA-sequencing and analysis. Subjects included persons referred to the ARC clinic for FEVR evaluation and some close relatives of FEVR or suspected FEVR patients. Many patients travelled from other US states or other countries, so an examination of all first-degree relatives by the ARC clinic was often not possible. After ARC clinical evaluation, 35/76 subjects were considered to have a confirmed FEVR phenotype and were graded for FEVR clinical staging as described previously by the ARC group [13,14]. Additionally, 20/76 subjects were clinically confirmed to be unaffected by FEVR, 5/76 subjects were considered to have potential mixed FEVR and ROP (FROP) (Retinopathy of Prematurity), 1/76 was clinically classified as just ROP, and 15/76 subjects were of unknown FEVR status. The latter subjects were mostly relatives of FEVR patients who provided blood samples to the ARC Eye Biobank but who could not be clinically evaluated for FEVR.

### 2.2. Sample Storage and Handling

Whole-blood samples were collected at the clinical site and frozen at −70 °C. Frozen aliquots (100–200 µL) of blood samples from consenting persons were made in 1.5 mL sterile microfuge tubes and kept frozen until use. All blood samples obtained from subjects were de-identified and analyzed with a randomly assigned research ID number prior to transportation to the Oakland University Eye Research Institute (ERI) with lists of sample IDs. The research side of the project (ERI) did not have access to any key for decoding sample identification. Family relationships between de-identified samples and their gender were known for analysis purposes only. Samples were stored at −70 °C in the ERI until extraction of genomic DNA.

### 2.3. Extraction of Genomic DNA

Genomic DNA was extracted from 100–200 µL of frozen whole blood with the Thermo-Fisher Pure-Link Genomic DNA Extraction Kit (ThermoFisher, Waltham, MA, USA). DNA concentrations were measured with a Qubit Fluorimeter, from 1 µL of genomic DNA extracted, with the Qubit dsDNA HS Assay Kit (ThermoFisher, Waltham, MA, USA).

### 2.4. AmpliSeq Targeted Library Design In Silico

The targeted AmpiSeq panel was designed in silico using Illumina’s DesignStudio (https://designstudio.illumina.com/, accessed on 12 January 2022). Parameters included eight target genes, with seven of the genes related to FEVR/ND and another very rare pediatric disease gene (*RS1*) for retinoschisis: *NDP* (ChrX), *RS1* (ChrX); *CTNNB1* (Chr3); *TSPAN12* (Chr7); *KIF11* (Chr10), *FZD4* (Chr11), *LRP5* (Chr11), *ZNF408* (Chr11). Options were selected for an average 250 base pair amplicon size, targeting exons with amplicon overlap, and at least 25 base pairs of 5-prime and 3-prime adjacent intron sequence. Designs of different pool number were compared in silico, and 3 pools were selected from predictions of >99.95% base coverage. The final Amplicon targeted panel numbered 360 PCR-primers to generate 180 amplicons, covering just over 32,000 base pairs in a sequence for 83 exons.

### 2.5. AmpliSeq Targeted Library Preparation

Illumina’s AmpliSeq Library preparation protocol was followed as per the manufacturer’s instructions. (Illumina, Sand Diego, CA, USA) Other Illumina reagents and kits were used per the Illumina workflow. The AmpliSeq Library Plus kit was used for 16 samples per kit using a three-pool amplicon set (beginning with three PCRs before combining into one PCR product per patient). The AmpliSeq CD Indexes Set B for Illumina (96) was used in the workflow to provide unique bar-coded end indexes for each patient library. These provided for a different index for each strand to enable separate sequencing for each paired amplicon strand.

Final targeted amplicon libraries were quality-checked using an Agilent Bioanalyzer and the Agilent DNA 1000 kit. After a few groups of libraries were found to always be of sufficient quality for sequencing, libraries could also simply be quantified using the Qubit DNA assay to establish library DNA concentration. In total, 16 to 48 libraries were diluted to equal concentrations according to Illumina’s protocol, and a 5% non-human PhiX DNA spike-in standard (Illumina) was included for monitoring of sequencing error rate and quality by the iSeq-100 system. Final total pooled library concentration was 50 pM, divided equally among samples (up to 50, of which 20 µL was loaded into a single-use iSeq-100 Reagent V2 cartridge and flow cell). Typical run times started in the afternoon, continuing overnight, and took 18–19 h from instrument start to the delivery of final variant call file data for all samples.

### 2.6. Sequencing on the iSeq-100 System

For sequencing, up to 48 patient sequencing libraries were pooled for a single instrument run. The on-board software workflow provided by the iSeq-100 included: Illumina’s DNA Amplicon Workflow (3.24.1.8+master), BWA-MEM Whole-Genome (Aligner, 0.7.9a-isis-1.0.2), Pisces Variant Caller (5.2.9.23), Illumina Annotation Engine (2.0.11-0-g7fb24a09), Bam Metrics (v.0.0.22), and SAMtools (0.1.19-isis-1.0.3). The annotation source option was selected as RefSeq, and the dataset was version 91.26.44. Options for sequencing included paired end sequencing format, with a minimum threshold read depth of 10 for final base calling. Reference genome selection was Homo Sapiens NCBI GRCh38 with decoys.

### 2.7. Bioinformatics Data and Analysis

The data from sequencing the eight genes involved in Norrie disease, FEVR, and retinoschisis typically provided about 20–30 DNA sequence variants per person using the reference human genome sequence (GRCh38 - hg38). Variant impacts on protein sequences and allele frequency data were determined from the ClinVar database (https://www.ncbi.nlm.nih.gov/clinvar/), the Genome Aggregation Database, gnomAD https://gnomad.broadinstitute.org/, and the Variant Effect Predictor (VEP) database at ENSEMBL (https://uswest.ensembl.org/info/docs/tools/vep/index.html). Databases were last accessed on 12 January 2022. Bar graphs and statistical testing were completed using software tools in R, managed in the RStudio Desktop open-source environment (Software available from https://www.rstudio.com).

## 3. Results

### 3.1. Sequencing Data Quality and Performance

Table 1 contains the sequencing quality metrics obtained in a multiple-sample run using the designed AmpliSeq targeted library. Overall, 95.5% of base reads were >Q30 quality (error < 1/1000). The percentage of on-target bases passing filter (PF) was 92.2%. Average sequencing depth of coverage was 978.

### 3.2. Total Variant Detection Results

Numerous variants were detected from the 76 persons tested. Table 2 contains the counts for SNVs (single-nucleotide variants), insertions, and deletions according to variant category and location. Average numbers of variants per sample were: 19.5 SNVs, 1.7 inserts, and 1.5 deletions. A total of 937 SNVs were detected, of which 3 SNVs were not found in the dbSNP database. Of a total of 81 insertions, 3 were not found in dbSNP, and of 71 deletions, 10 were not present in dbSNP.

### 3.3. Protein-Altering Variants in Cohort of 76 FEVR Subjects and Relatives

**Figure 2:**
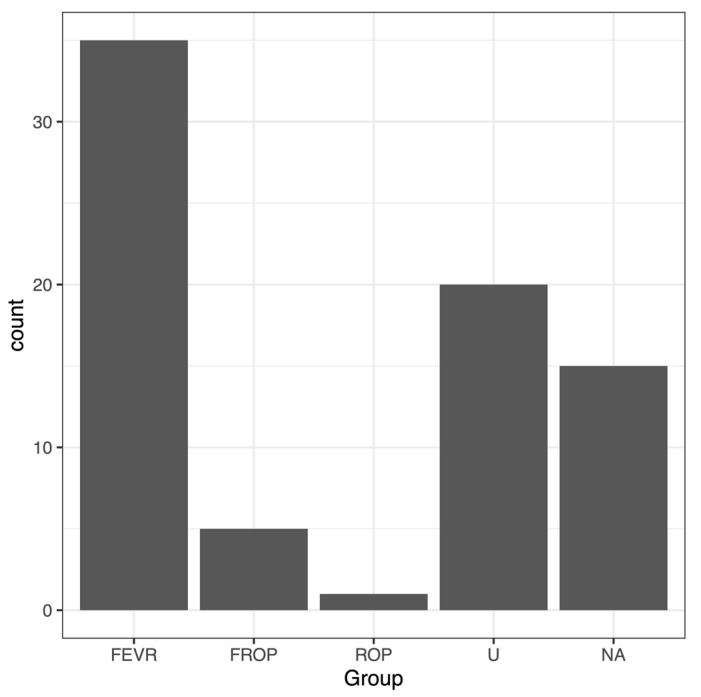
Final group distribution of 76 sequenced subjects. The largest grouping contained patients confirmed in the ARC clinic with FEVR (35). ARC confirmed FEVR-Unaffected subjects (U) numbered 20. Five pediatric subjects were categorized as ROP with potential FEVR complication (FROP), while one pediatric subject was ROP. Fifteen subjects, mostly relatives of FEVR patients, were not available to ARC for retinal diagnosis (NA).

Persons included in this round of sequencing were refered to Associated Retinal Consultants (ARC), and age, diagnosis, and any family relationships at presentation to the ARC clinic are listed in Table 3. The sample set included related persons from 18 families grouped first in the table, followed by individual patients without relatives. (Note: there is no Family 6.) FEVR stages (base-grade) are provided in Table 3 if stages were confirmed by examination at the ARC clinic. The base stages were: (1) avascular periphery, (2) retinal neovascularization present, (3) extramacular retinal detachment, (4) macula-involving retinal detachment, and (5) total retinal detachment. The final distribution of subjects grouped into five categories is shown in Figure 2: FEVR, U (Unaffected by FEVR), ROP (Retinopathy of Prematurity), ROP with possible FEVR complication (FROP), and NA (unknown, Not Available).

Of the entire cohort sequenced, 50 of the subjects were present with a close relative, representing 18 family groupings. Other subjects in the cohort were included as unrelated individuals who were referred to the ARC clinic for potential diagnosis of FEVR. Results for the family groupings are summarized below. Note: there was no Family 6 designation in this study.

**Figure 3:**
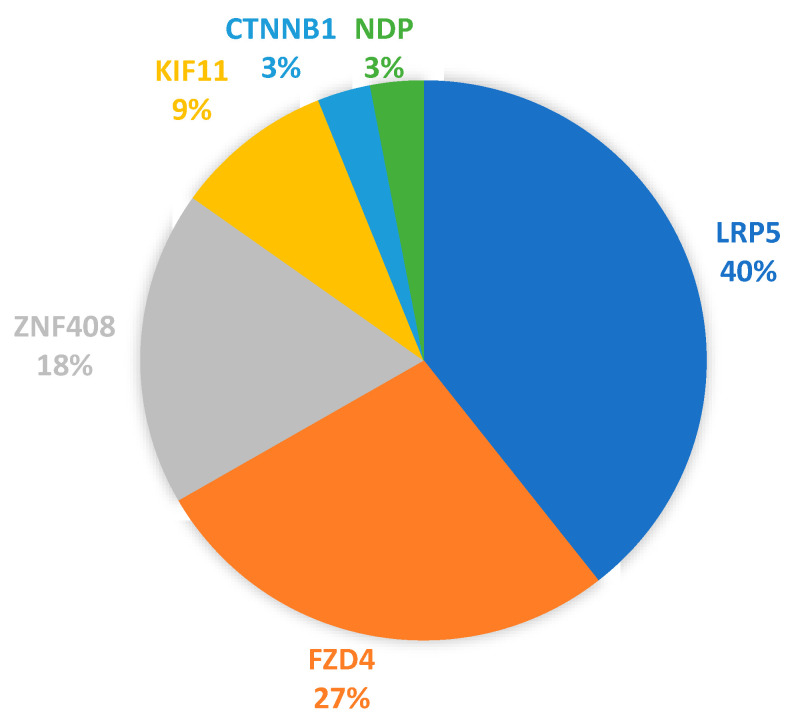
Gene distribution of protein sequence variants detected in the FEVR cohort. Of the 33 protein-altering variants listed in Table 4, the distribution by gene was as follows: *LRP5* 13/33 (40%), *FZD4* 9/33 (27%), *ZNF408* 6/33 (18%), (*KIF11* 3/33 (9%), *NDP* 1/33 (3%), *CTNNB1* 1/33 (3%).

Thirty-three (33) protein-altering variants were detected in the entire group of sequenced patients. These variants with their HGVS descriptions are listed in Table 4, as well as any known fractional allele frequencies (AF). Of the 33 variants, 85% were found in just three of the seven genes: *FZD4, LRP5*, and *ZNF408*. Figure 3 contains a chart of the relative variant-to-gene distributions: *LRP5* 13/33 (40%), *FZD4* 9/33 (27%), *ZNF408* 6/33 (18%), *KIF11* 3/33 (9%), *NDP* 1/33 (3%), *CTNNB1* 1/33 (3%). The impacts on protein sequences were: 27 single amino substitutions (81%), 3 amino acid deletions (9%), 1 stop-gained alteration (3%), 1 amino acid insertion duplication (3%), and 1 splice donor site loss (3%).

Two novel, likely pathogenic variants were detected in one family and one unrelated individual based upon their similarity to known pathogenic variants that truncate significant portions of FZD4 and LRP5. FZD4: Cys450ter (Family 11) and LRP5: Ala919CysfsTer67 (Subject 59). Four previously known pathogenic protein-altering variants were found in five families and two unrelated individuals. They included: FZD4: Met105Val (Family 7, 9, 11), LRP5: c.4488+2T>G (splice donor) (Family 10, 14), LRP5: Cys913LeufsTer73 (Subject 58), and NDP: His42Arg (Subject 56) (Table 3).

Family 1: Affected twins both had LRP5: Val667Met and LRP5: Ala1330Val with high FEVR grades. The LRP5: Ala1330Val Allele Frequency (AF) is 13.4%, currently categorized in ClinVar as likely benign. LRP5: Val667Met is also considered benign (AF = 13.4%) Interestingly, Proband-1 has more severe disease (grade 5) than their twin (grade 4) and the LRP5: Ala1330Val homozygous variant, while their twin is heterozygous for both variants. The visual status of the mother, maternal aunt, and maternal uncle were not known.

Family 2: Both the proband son and the mother had lower FEVR grades, but they had different protein-altering variants. The proband’s variant CTTNB1: Val273Met has a very low Allele Frequency (AF = 0.0021%), was not found in ClinVar and would be of uncertain significance at this time. The mother had digenic variants that are listed as benign in ClinVar, LRP5: Leu20dup and ZNF408: Arg337Pro.

Family 3: The LRP5: Ala1330Val homozygous variant was combined with the LRP5: Leu20dup benign variant in proband-3, an adult son with a FEVR grade of 4. The retinal status of the proband’s father was unknown, but the father was heterozygous for he LRP5: Ala1330Val variant.

Family 4: Sequencing was only possible for the the parents of an affected, not-yet-sequenced infant, who were themselves unaffected by FEVR. Only the father had a combination of two currently likely benign and benign variants: LRP: Met1086Val and ZNF408: Arg337Pro.

Family 5: The proband infant daughter (FEVR grade 3) shared the same two individually benign variants in LRP5 and ZNF408 as her unaffected mother. The father had a FEVR base-grade 1 and the same ZNF408 variant but two completely different likely benign and benign variants of LRP5.

Family 7: A known pathogenic variant FZD4: Met105Val was present in the proband teenage daughter with a FEVR base-grade 2. Clinical diagnosis of the father, mother, and one half-sibling was not available. The father had the same FZD4: Met105Val pathogenic variant, while the mother had no detected protein changes. The related sister did not have the pathogenic variant, only one benign variant (ZNF408: Val194_Val197del) inherited from the father.

Family 8: The proband teenage daughter diagnosed with FEVR grade 1 and two diagnosed unaffected pediatric siblings were sequenced. The proband had two variants. One, KIF11: Glu129Ala, of low allele frequency < 0.0001, not found in ClinVar, we considered to be of uncertain significance. The second variant present was homozygous, LRP5: Ala1330Val, a variant categorized as benign in ClinVar. The LRP5: Ala1330Val variant was present and heterozygous in an unaffected brother. An unaffected sister had the relatively common and benign variant ZNF408: Val194_Val197del (AF = 13.4%).

Family 9: The proband pediatric son had the known pathogenic variant FZD4: Met105Val (AF = 0.0043%), correlating with a severe FEVR base-grade of 5. Sequencing was completed for the unaffected father, who was found to have no protein changes from the panel. Presumably the pathogenic variant was inherited maternally.

Family 10: The proband was a young adult female, 18 years of age at presentation to our clinical group, with a FEVR base-grade of 5. A splice donor variant in LRP5: c.4488+2T>G considered to be pathogenic was detected in the proband and in the mother and a maternal uncle. Both the mother and uncle presented with a FEVR grade of 1. This variant was not inherited by the uncle’s son, who is unaffected and was found to have no protein changes from the panel. The mother had one additional variant, LRP5: Leu20dup (AF = 10.1%), considered benign. The proband also had a second variant, FZD4: Pro11Gln (AF = 0.0021%), not in ClinVar that we currently consider of uncertain significance.

Family 11: The proband pediatric daughter had a FEVR grade of 5 at presentation to the clinic and was found to have two known pathogenic variants, FZD4: Cys450ter and FZD4: Met105Val. The first variant FZD4: Cys450ter was presumably inherited from the mother, who had a FEVR grade of 1. The father was not available for sequencing. The mother had a second variant, the benign and common LRP5: Leu20dup, which was also detected in the proband’s maternal grandfather, who was unaffected. The proband’s maternal grandfather also had a second common benign variant, ZNF408: Val194_Val197del (AF = 13.1%).

Family 12: The proband, pediatric daughter had a base FEVR grade of 5 and two LRP5 variants considered benign in ClinVar: LRP: Val667Met and LRP: Ala1330Val. The retinal status of the parents was not known. Both variants appeared to be inherited from the father, who also had a third common variant, ZNF408: Val194_Val197del. The mother had no protein changes from the panel.

Family 13: The proband pediatric son with a FEVR grade of 4 was found to have the same two benign LRP5 variants as in Family 12. LRP: Val667Met and LRP: Ala1330Val. The proband’s mother with a FEVR grade of 1 had no protein-altering variants from the panel. The same two variants were shared by an unaffected sister and were inherited from the unaffected father who also had the common variant ZNF408: Val194_Val197del. The maternal grandmother was unaffected and had no protein changes from the test panel, while the maternal grandfather with a FEVR base grade of 1 had four common benign variants, LRP5: Val667Met, LRP5: Ala1330Val, FZD4: Pro33Ser, and FZD4:Pro168Ser.

Family 14: The proband, pediatric son with FEVR grade 4 was found to have the pathogenic LRP5: c.4488+2T>G splice donor variant. The mother with a FEVR grade of 1 did not have this variant but was found have a common variant, ZNF408: Val194_Val197del (AF = 13%), and a deletion, LRP5: Leu16_Leu20del (AF = 0.0007%), which was not in ClinVar and we currently categorize as of uncertain significance.

Family 15: Currently, two twin boys were sequenced with base FEVR grades of 1 and 2 at the time of presentation. Both had the same variant ZNF408: Glu230Gly, listed in ClinVar as likely benign (AF = 0.037%).

Family 16: The proband was a male pediatric case referred with possible FEVR but subsequently considered unaffected by FEVR. One sister without a clinical work up was also sequenced. Both had two common benign variants, LRP5: Ala1330Val and ZNF408: Val194_Val197del.

Family 17: The proband was an infant with uncertain FEVR, whose sample was not available for sequencing. Both parents of unknown retinal status were sequenced and found to have a common benign deletion variant, ZNF408: Val194_Val197del (AF = 13%).

Family 18: The proband was an infant with severe bilateral FEVR with retinal detachment whose sample was not available for sequencing. The mother is diagnosed as unaffected by FEVR, and no missense variants were detected upon sequencing. The father’s retinal status is unknown, but DNA sequencing detected two common benign variants, LRP5: Val667Met and LRP5: Ala1330Val, and a more rare variant, FZD4: Arg127Cys (AF = 0.0046%), that was listed as likely benign in ClinVar.

### 3.4. Multiple Variants and Multigenic Variants

**Figure 4:**
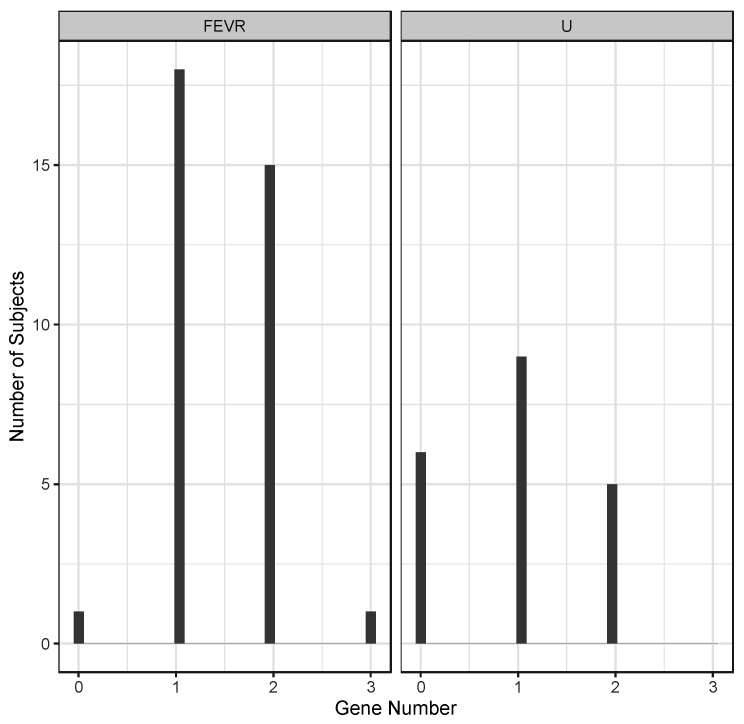
Distribution of the number of affected genes with protein-altering variants in affected and unaffected FEVR subjects. Numbers of genes are shown for 35 FEVR subjects and 20 Unaffected (U) subjects as confirmed by ARC diagnosis.

Data from all the subjects sequenced revealed the existence of multiple protein variants in single genes, as well as variants in multiple FEVR-related genes in the same individuals. The latter included both digenic and trigenic variant combinations, as summarized in Table 5. Considering just the subset of patients with an ARC-confirmed diagnosis of FEVR or Unaffected (U), the distribution of the number of genes with protein-altering variants is shown in Figure 4.

The average number of genes with protein-altering variants in the group of confirmed FEVR diagnosis was greater than the unaffected group of subjects, averaging 1.46 genes for FEVR and 0.95 genes for Unaffected (*p* = 0.009, *t*-test). Of the total subjects sequenced in this cohort, 26/76 were di- or tri-genic for protein-altering variants within the group of 7- FEVR-linked genes sequenced. Using data from the GnomAD database, derived from over 14,000 genome sequences, the occurrence of the same combined frequencies in the general population was estimated to total 3.6%. This was substantially lower than the frequency in our cohort, 34.2% (Chi-squared, *p* = 3.0 × 10^−17^).

## 4. Discussion

### 4.1. FEVR Phenotype and Genes

While numerous genes are involved in the FEVR phenotype, there is no simple correlation between disease severity and specific genes. This is most likely because the impact on the neural retina results from an avascular peripheral retina that results in hypoxia and elevated vascular endothelial growth factor. Any genetic variant that inhibits development of the neural retinal vasculature will generate the situation of peripheral hypoxia. Similar to ROP (retinopathy of prematurity), the smaller the final zone of vascularized retina, the more severe the depth of hypoxia and the greater the potential for a more severe FEVR phenotype [1].

The Norrin-Wnt signaling pathway is unique to the neural retinal endothelium, and indeed, active Norrin signaling is essential for normal growth and proliferation of the developing retinal vasculature [7,15]. Likewise, a loss of KIF11′s kinesin activity can impair growth and proliferation of the retinal vasculature and again results in peripheral avascular neural retina [9]. KIF11 FEVR patients tend to have other significant syndromes (MLCRD, CDMMR), none of which were noted in the 76 subjects presented for analysis here. ZNF408 is a multiple zinc-finger DNA-binding protein, and point mutations that impair ZNF408′s DNA-binding activity result in diminished expression of genes required for vascular development [16]. Thus, disrupting the normal function of Norrin-Wnt signaling, KIF11 kinesin action, or ZNF408′s gene activation activity can result in mild to severe FEVR phenotypes.

An AmpliSeq targeted-sequencing panel including seven FEVR-related genes revealed protein-altering variants in most of the subjects that were sequenced for this initial study. The cohort included persons diagnosed with FEVR, close relatives of some proband FEVR patients, and individual persons referred to the ARC clinic for potential FEVR. Of the 33 protein-altering variants detected, 85% were found in three of the genes: *FZD4, LRP5*, and *ZNF408*. *ZNF408* ranked third in the number of protein-changing variants we found in this study, which was also found in one small study from China [6]. Another study from China of a larger cohort of several hundred *FEVR* patients found that *TSPAN12* variants ranked third after *FZD4* and *LRP5* [17]. *FZD4* and *LRP5* variants were the two most frequent in our cohort, which seems to be a distribution shared with previously published studies [6,17,18,19]. Together, these studies indicate some regional variation occurs, but in general, the two most commonly variant genes were *FZD4* and *LRP5.* Most of the variants, 20/33, had low allele frequencies, <0.01%, and are candidate variants to be analyzed further for their potential contribution to FEVR in this group of subjects.

### 4.2. Frequency of Multigenic Protein-Altering Variants

Our targeted panel covered seven FEVR-related genes with over 900 times average read coverage and >95.5% Q-30 quality reads, which provided us with high confidence in sensitivity for variant detection and for determination of zygosity. These sequencing results have permitted us to answer the question posed in the introduction: what percentage of FEVR subjects may have multiple protein-altering variants in this pool of seven FEVR-related genes? In our cohort, 34% (26/76) of subjects had multiple protein-altering variants in two or three different FEVR-related genes. This was greater than the 3.6% expected from the general population, based on GnomAD genome data. Selections of patients and families based on FEVR phenotypes greatly increased the relative presence of multigenic protein-altering variants in the FEVR-related gene set. While this association is not sufficient proof that multigenic variants contribute to the FEVR phenotype, the high percentage of multigenic variants indicates that we cannot absolutely rule out such potential contributions due to scarcity.

Li et al. [6] reported detection of potential digenic variants in just under 3% of their study cohort when sequencing four FEVR-related genes (*FZD4*, *LRP5*, *TSPAN12*, *ZNF408*) [6]. A relatively higher incidence of multigenic variants in our study may be due to several factors, including the inclusion of more FEVR-related genes, sequencing to a greater depth for sensitivity, and the fact that ZNF408 variants were relatively highly represented in our cohort. ZNF408 variants occurred in 18 subjects in digenic and trigenic combinations.

Comparing only the confirmed FEVR-positive to confirmed FEVR-negative (unaffected) individuals in our cohort, we found that the average number of FEVR-related genes per person with protein-altering variations was higher in the confirmed FEVR subset. While this also was not sufficient proof to conclude that multigenic contributions are important in FEVR incidence, this relative occurrence would be expected in that scenario. Some of our specific findings have also provided us with guidance for continued follow-up of specific families, where we can extend pedigrees with both sequencing and clinical diagnosis.

### 4.3. Known and Novel Pathogenic Variants

Pathogenic variants were uncovered in several families (Table 3 and Table 4). FZD4: Met105Val was found in Family 7, 9, and 11. A new likely pathogenic variant, FZD4: Cys450ter, was also found in Family 11. The splice donor variant *LRP5*: c.4488+2T>G was detected in Family 10 and 14. The other pathogenic variants detected in individual subjects included LRP5: Cys913LeufsTer73 and a new similar frame shift termination variant LRP5: Ala919CysfsTer67 (likely pathogenic). Finally, one known pathogenic variant affecting Norrin itself is NDP: His42Arg.

Family 1 leads us to consider an additional question going forward. Can heterozygous variants currently considered benign on their own contribute to FEVR occurrence when homozygous or when two different variants impact the same gene such as LRP5? That situation may be present here, where both twins had two variants in LRP5: Val 667Met and Ala1330Val but the more severely affected twin inherited the LRP5: Ala1330Val variant homozygous. Interestingly, this homozygous variant LRP5: Ala1330Val also occurred in the Family 3 proband, again in combination with a different LRP5 benign variant. Again, LRP5: Ala1330Val appeared homozygous in the Family 8 proband with a novel KIF11 variant of unknown significance, while the LRP5: Ala1330Val variant was present and heterozygous in an unaffected sibling.

Family 10 was identified for future analysis of a potential digenic contribution to FEVR severity. Both the mother and uncle of proband10 had a base FEVR grade of 1 and shared a pathogenic splice variant in LRP5: c.4488+2T>G. The proband was more severely affected with FEVR (stage 5) and had a second variant of uncertain significance in FZD4: Pro11Gln (AF = 0.0021%). The uncle’s son, without any of these variants, was unaffected by FEVR.

Another example of features that can complicate our understanding of FEVR severity within the same family is the occurrence of completely different variants, with some present in a multigenic manner. Family 14 was identified for future follow-up analysis as an example of this situation. The proband son had a FEVR base grade of 4 and the *LRP5* pathogenic splice donor variant, *LRP5*: c.4488+2T>G, while the proband’s mother has a lower FEVR grade of 1 but did not have this variant. Instead, the mother was digenic for the benign ZNF408: Val194_Val197del (AF = 13%) and LRP5: Leu16_Leu20del (AF = 0.0007%). The latter was not in ClinVar, and we currently categorize this variant as of uncertain significance.

### 4.4. Sequencing Approach

Targeted sequencing, Whole-Exome Sequencing (WES), and Whole-Genome Sequencing (WGS) have all been employed for genetic analysis of Inherited Retinal Diseases (IRDs). All three have their advantages and disadvantages. A large IRD targeted panel applied to a disease-appropriate cohort of 85 children demonstrated 80% identification of the likely disease-causing variant [20]. Governed by the concept that “perfection is the enemy of progress” (Winston Churchill, speech, 11 October 1952), we designed a customized seven-gene targeted panel, to balance several parameters including the patient consenting process, FEVR-gene sequencing coverage, SNV sensitivity, and cost.

Targeted IRD disease panels exist that include some FEVR-related genes, but they are missing one or more key FEVR-linked genes. While WES can survey most of the known exome, some IRD gene comparison studies have shown that targeted sequencing of IRD genes is generally superior to WES, as the latter may have poor capture efficiency for some exome regions [21,22]. WGS sequencing is theoretically the best method to survey all genes without a predetermined bias, but the ability to improve accuracy may require sequencing to a depth of coverage that greatly reduces the number of patients that can be tested with the available resources. While genome-wide variant data can contain a potentially novel disease-linked variant, a logical functional analysis may not be available to identify which variants from tens of thousands are relevant to the disease process.

Another consideration when adopting the WES and WGS approach concerns the potential detection of non-ocular genetic disease variants. In the United States, the American College of Medical Genetics and Genomics (ACMG) recommends that additional protocols should be included for reporting a pathological variant from the current ACMG list of medically actionable genes [23]. Currently, medical actionable gene reporting is not policy in most countries. Full patient education for ethical consent is even more important, and the choice to participate is more complicated for families considering the potential discovery of serious non-ocular health conditions using genome-wide analysis.

## 5. Conclusions

From this initial study, we can now plan future analysis of individual probands and additional relatives to determine if any of the family-specific multigenic variant combinations might influence phenotype penetrance in FEVR. The initial question posed by this study was answered: what is the incidence of multigenic protein-altering variants among known FEVR-linked genes in our FEVR cohort? In this study, the answer was 34%, far exceeding the percentage expected in the general population. This high proportion of multigenic protein-altering variants in a specific FEVR-linked gene panel establishes that there is a theoretical potential for multigenic variants to contribute to FEVR’s variable phenotypic presentation. While we cannot point to absolute proof of multigenic contributions to the FEVR phenotype from this initial study, this data set forms the basis for further investigation of this possibility.

## Figures and Tables

**Table 1:** Sequencing Parameters. General sequencing metrics as obtained with 48 pooled samples on a single iSeq-100 run.

Parameter	Value
Number of amplicon regions	180.0
Total length of target regions	32,731.0
Percent on-target aligned reads	98.3
Percent on-target PF reads	92.9
Percent aligned read	94.6
Percent on-target aligned bases	98.4
Percent on-target PF bases	92.2
Percent Q30 bases	95.5
Percent aligned bases	93.7
Amplicon mean coverage	978.0

**Table 2:** Variant types. Variants detected, by type, from the sequencing of 48 pooled sequencing library samples with the 8-gene panel. Metrics for SNVs (single nucleotide variants), insertions, and deletions are shown as total counts and the average count per sample.

Variant Type	Total Count	Average per Sample
SNVs total	937	19.5
SNVs in genes	927	19.3
SNVs in exons	268	5.6
SNVs in coding regions	220	4.6
SNVs in UTR regions	48	1.0
SNVs in splice site regions	55	1.1
Stop-gained SNVs	2	0.04
Stop-lost SNVs	0	0.0
Non-synonymous SNVs	44	0.9
Synonymous SNVs	174	3.6
SNVs not in dbSNP	3	0.1
Insertions total	81	1.7
Insertions in genes	28	0.6
Insertions in exons	10	0.2
Insertions in coding regions	8	0.2
Insertions in UTR regions	2	0.04
Insertions in splice site regions	0	0.0
Stop-gained insertions	0	0.0
Stop-lost insertions	0	0.0
Frame shift insertions	2	0.04
Non-synonymous insertions	6	0.1
Insertions not in dbSNP	3	0.1
Deletions total	71	1.5
Deletions in genes	26	0.5
Deletions in exons	19	0.4
Deletions in coding regions	15	0.3
Deletions in UTR regions	4	0.1
Deletions in splice site regions	0	0.0
Stop-gained deletions	0	0.0
Stop-lost deletions	0	0.0
Frame shift deletions	0	0.0
Non-synonymous deletions	15	0.3
Deletions not in dbSNP	10	0.2

**Table 3:** Protein-altering variants from targeted sequencing of 76 subjects. Patients were referred to the clinic with FEVR diagnosis or to evaluate potential FEVR status. Some close relatives of FEVR subjects were also sequenced. FEVR grade at clinical presentation is shown for confirmed FEVR group subjects. Other group notations are: U (Unaffected, confirmed not-FEVR), ROP (Retinopathy of Prematurity), FROP (FEVR/ROP, potential ROP overlapping with FEVR but not confirmed FEVR), and Not Available (NA). Known FEVR-causing pathogenic variants, likely pathogenic variants, and their corresponding Allele Frequencies (AF) are shown in **bold**-face type. Allele frequency estimates from the GnomAD data base are provided as well as the estimated frequency of combinations. Subjects with family members referred to clinic are grouped at the top of the table by family designation. A summary listing of the coding variants in HGVS format is provided in Table 4. (Note: there was no Family #6 in this FEVR-based study).

	Group	FEVR Grade	Family	Gender	Age	Relationship	Coding-Variations	AF 1	AF 2	AF 3	AF 4	Combined AF
1	NA	NA	1	M	43	maternal uncle of 4 and 5	LRP5:Val 667Met LRP5:Ala1330Val	0.034	0.134	NA	NA	0.004556
2	NA	NA	1	F	44	maternal aunt of 4 and 5	LRP5:Val 667Met LRP5:Ala1330Val	0.034	0.134	NA	NA	0.004556
3	NA	NA	1	F	41	mother of 4 and 5	LRP5:Val 667Met LRP5:Ala1330Val	0.034	0.134	NA	NA	0.004556
4	FEVR	5	1	M	1	pediatric, son, proband 1	LRP5:Val 667Met LRP5:Ala1330Val (homo)	0.034	0.016	NA	NA	0.000544
5	FEVR	4	1	M	1	pediatric, twin of 4	LRP5:Val 667Met LRP5:Ala1330Val	0.034	0.134	NA	NA	0.004556
6	FEVR	1	2	F	42	mother of 7	LRP5: Leu20dup ZNF408:Arg337Pro	0.1014	0.0086	NA	NA	0.000872
7	FEVR	2	2	M	1	pediatric, son, proband 2	CTTNB1:Val273Met	0.000021	NA	NA	NA	0.000021
8	NA	NA	3	M	67	father of 9	LRP5:Ala1330Val	0.134	NA	NA	NA	0.134
9	FEVR	4	3	M	29	son, proband 3	LRP5:Leu20dup LRP5:Ala1330Val (homo)	0.1014	0.016	NA	NA	0.001622
10	U	0	4	F	28	mother of pediatric not collected yet	LRP5: Met1086Val ZNF408:Arg337Pro	0.002077	0.0086	NA	NA	0.000018
11	U	0	4	M	30	father of pediatric not collected yet	No protein changes	NA	NA	NA	NA	NA
12	U	0	5	F	42	mother of 14	LRP5:Leu20dup ZNF408:Val194_Val197del	0.1014	0.1307	NA	NA	0.013253
13	FEVR	1	5	M	54	father of 14	LRP5:Val667Met LRP5:Ala1330Val ZNF408:Val194_Val197del	0.034	0.134	0.1307	NA	0.000595
14	FEVR	3	5	F	3	pediatric, daughter, proband-5	LRP5:Leu20dup ZNF408:Val194_Val197del	0.1014	0.1307	NA	NA	0.013253
15	NA	NA	7	M	45	father of 17 and 18	**FZD4:Met105Val** LRP5:Ala1330Val ZNF408:Val194_Val197del	**0.000043**	0.134	0.1307	NA	0.000001
16	NA	NA	7	F	43	mother of 17	No protein changes	NA	NA	NA	NA	NA
17	FEVR	2	7	F	16	pediatric, daughter, proband-7	**FZD4:Met105Val** LRP5:Ala1330Val	**0.000043**	0.134	NA	NA	0.000006
18	NA	NA	7	F	19	sibling of 17	ZNF408:Val194_Val197del	0.1307	NA	NA	NA	0.1307
19	FEVR	1	8	F	14	pediatric, daughter, proband-8	KIF11:Glu129Ala LRP5:Ala1330Val (homo)	0	0.016	NA	NA	0
20	U	0	8	F	11	pediatric, sibling of 19	ZNF408:Val194_Val197del	0.1307	NA	NA	NA	0.1307
21	U	0	8	M	13	pediatric, sibling of 19	LRP5:Ala1330Val	0.134	NA	NA	NA	0.134
22	U	0	9	M	31	father of 23	No protein changes	NA	NA	NA	NA	NA
23	FEVR	5	9	M	1	pediatric, son, proband-9	**FZD4:Met105Val**	**0.000043**	NA	NA	NA	0.000043
24	FEVR	1	10	M	57	maternal uncle of 26	**LRP5:c.4488+2T>G (splice donor)**	0	NA	NA	NA	0
25	FEVR	1	10	F	45	mother of 26	**LRP5:c.4488+2T>G (splice donor)** LRP5:Leu20dup	0	0.1014	NA	NA	0
26	FEVR	5	10	F	18	daughter, proband-10	**LRP5:c.4488+2T>G (splice donor)** FZD4:Pro11Gln	0	0.000021	NA	NA	0
27	U	0	10	M	17	son of maternal uncle 24	No protein changes	NA	NA	NA	NA	NA
28	U	0	11	M	68	maternal grandfather of 30	LRP5:Leu20dup ZNF408:Val194_Val197del	0.1014	0.1307	NA	NA	0.013253
29	FEVR	1	11	F	30	mother of 30	**FZD4:Cys450ter** LRP5:Leu20dup	**0**	0.1014	NA	NA	**0**
30	FEVR	5	11	F	3	pediatric, daughter, proband-11	**FZD4:Cys450ter FZD4:Met105Val**	**0**	**0.000043**	NA	NA	**0**
31	NA	NA	12	M	28	father of 33	LRP5:Val667Met LRP5:Ala1330Val ZNF408:Val194_Val197del	0.034	0.134	0.1307	NA	0.000595
32	NA	NA	12	F	26	mother of 33	No protein changes	NA	NA	NA	NA	NA
33	FEVR	5	12	F	1	pediatric, daughter, proband-12	LRP5:Val667Met LRP5:Ala1330Val	0.034	0.134		NA	0.004556
34	NA	NA	13	F	74	grandmother of 38	No protein changes	NA	NA	NA	NA	NA
35	FEVR	1	13	M	73	grandfather of 38	LRP5:Val667Met LRP5:Ala1330Val FZD4:Pro33Ser FZD4:Pro168Ser	0.034	0.134	0.01236	0.014077	0.000001
36	U	0	13	M	49	father of 39	LRP5:Val667Met LRP5:Ala1330Val ZNF408:Val194_Val197del	0.034	0.134	0.1307	NA	0.000595
37	FEVR	1	13	F	47	mother of 38	No protein changes	NA	NA	NA	NA	NA
38	FEVR	4	13	M	11	pediatric, son, proband-13	LRP5:Val667Met LRP5:Ala1330Val	0.034	0.134	NA	NA	0.004556
39	U	0	13	F	12	pediatric, daughter, sibling of 38	LRP5:Val667Met LRP5:Ala1330Val	0.034	0.134	NA	NA	0.004556
40	FEVR	1	14	F	46	mother of 41	LRP5:Leu16_Leu20del ZNF408:Val194_Val197del	0.000007	0.1307	NA	NA	0.000001
41	FEVR	4	14	M	6	pediatric, son, proband-14	**LRP5:c.4488+2T>G (splice donor)**	0	NA	NA	NA	0
42	FEVR	2	15	M	3	pediatric, son, twin of 43	ZNF408:Glu230Gly	0.000371	NA	NA	NA	0.000371
43	FEVR	1	15	M	3	pediatric, son, twin of 42	ZNF408:Glu230Gly	0.000371	NA	NA	NA	0.000371
44	NA	NA	16	F	8	pediatric, daughter, sibling of 47	LRP5:Ala1330Val ZNF408:Val194_Val197del	0.134	0.1307	NA	NA	0.017514
45	U	0	16	M	11	sibling of 46	LRP5: Ala1330Val ZNF408:Val194_Val197del	0.134	0.1307	NA	NA	0.017514
46	NA	NA	17	M	NA	father of child (FEVR not sequenced)	ZNF408:Val194_Val197del	0.1307	NA	NA	NA	0.1307
47	NA	NA	17	F	37	mother of child (FEVR not sequenced)	ZNF408:Val194_Val197del	0.1307	NA	NA	NA	0.1307
48	NA	NA	18	M	41	father of child (FEVR not sequenced)	FZD4:Arg127Cys LRP5:Val667Met LRP5:Ala1330Val	0.000046	0.034	0.134	NA	0
49	U	0	18	F	38	mother of child (FEVR not sequenced)	No protein changes	NA	NA	NA	NA	0
50	NA	NA	19	F	60	mother of child (FEVR not sequenced)	LRP5:Pro6Thr ZNF408:Val194_Val197del	0.002932	0.1307	NA	NA	0.000383
51	FEVR	3	NA	F	2	pediatric, no relations	LRP5:Val667Met LRP5:Ala1330Val LRP5:Pro848Leu LRP5:Thr852Met	0.034	0.134	0	0.000013	0
52	FEVR	1	NA	F	90	adult, no relations	KIF11:His526Gln ZNF408:Val194_Val197del	0.002532	0.1307	NA	NA	0.000331
53	FEVR	2	NA	M	11	pediatric, no relations	LRP5:Ala1330Val ZNF408:Val194_Val197del(homo)	0.134	0.0159	NA	NA	0.002131
54	FEVR	4	NA	F	6	pediatric, no relations	LRP5:Leu20dup ZNF408:Val194_Val197del	0.1014	0.1307	NA	NA	0.013253
55	FEVR	1	NA	F	54	adult, no relations	LRP5:Ala1330Val	0.134	NA	NA	NA	0.134
56	FEVR	4	NA	M	9	pediatric, no relations	**NDP:His42Arg**	**0.000005**	NA	NA	NA	**0.000005**
57	FEVR	2	NA	M	1	pediatric, no relations	LRP5:Val667Met LRP5:Ala1330Val ZNF408:Val194_Val197del	0.034	0.134	0.1307	NA	0.000595
58	FEVR	5	NA	F	1	pediatric, no relations	**LRP5:Cys913LeufsTer73**	**0.000004**	NA	NA	NA	**0.000004**
59	FEVR	5	NA	F	1	pediatric, no relations	**LRP5:Ala919CysfsTer67**	**0**	NA	NA	NA	**0**
60	FEVR	1	NA	M	14	pediatric, no relations	LRP5:Leu20dup	0.1014	NA	NA	NA	0.1014
61	FEVR	5	NA	M	1	pediatric, no relations	LRP5:Pro1522Leu FZD4:Ala408del	0.000291	0	NA	NA	0
62	FEVR	3	NA	F	34	adult, no relations	LRP5:Val667Met LRP5:Ala1330Val ZNF408:Val194_Val197del FZD4 Gly161Arg	0.034	0.134	0.1307	0	0
63	FROP	2	NA	M	2	pediatric, no relations	No protein changes	NA	NA	NA	NA	NA
64	FROP	NA	NA	F	1	pediatric, no relations	ZNF408:Val194_Val197del	0.1307	NA	NA	NA	0.1307
65	FROP	5	NA	F	1	pediatric, no relations	LRP5:Gln89Arg	0.008262	NA	NA	NA	0.008262
66	FROP	5	NA	F	1	pediatric, no relations	No protein changes	NA	NA	NA	NA	NA
67	FROP	4	NA	F	39	adult, no relations	LRP5:Ala1330Val	0.134	NA	NA	NA	0.134
68	U	0	NA	M	1	pediatric, no relations	LRP5:Leu20dup	0.1014	NA	NA	NA	0.1014
69	U	0	NA	M	7	pediatric, no relations	ZNF408:Val194_Val197del	0.1307	NA	NA	NA	0.1307
70	ROP	NA	NA	F	1	pediatric, no relations	ZNF408:Val194_Val197del FZD4:Trp139Ser	0.1307	0	NA	NA	0
71	U	0	NA	F	9	adult, no relations	LRP5:Leu20dup	0.1014	NA	NA	NA	0.1014
72	U	0	NA	F	15	pediatric, no relations	No protein changes	NA	NA	NA	NA	NA
73	U	0	NA	M	14	pediatric, no relations	KIF11:Pro642Ala	0.000293	NA	NA	NA	0.000293
74	U	0	NA	F	69	adult, no relations	LRP5:Leu20dup	0.1014	NA	NA	NA	0.1014
75	U	0	NA	M	43	adult, no relations	No protein changes	NA	NA	NA	NA	NA
76	U	0	NA	F	6	pediatric, no relations	ZNF408:Leu67Val ZNF408:Ala372Thr ZNF408:Pro647Gln	0.00368	0.000577	0	NA	0

**Table 4:** Variants altering the primary amino acid sequence. For six of the seven FEVR genes sequenced (*NDP*, *CTNNB1*, *KIF11*, *FZD4*, *LRP5*, *ZNF408*), thirty-three variants were detected that alter the primary amino acid sequence. Only synonymous variants were found in *TSPAN12*. Seven new variants are noted, including a likely pathogenic Cys450Ter stop-gained variant in FZD4. Allele Frequency (AF %) was derived from GnomDB. Accession numbers in dbSNP and ClinVar are shown where available. Consequence categories were from ClinVar, except those listed in parentheses, which are the current determinations of the authors.

No.	Gene	Nucleotide	Protein	Molecular Change	Consequence	AF %	AF (Homo) %	dbSNP	ClinVar Accession
1	CTNNB1	NM_001098209.2:c.817G>A	NP_001091679.1:p.Val273Met	missense variant	(Uncertain significance)	0.0021%	0.0%	rs1183899293	Not in ClinVar
2	FZD4	NM_012193.4:c.32C>A	NP_036325.2:p.Pro11Gln	missense variant	(Uncertain significance)	0.0021%	0.0%	rs766393047	Not in ClinVar
3	FZD4	NM_012193.4:c.97C>T	NP_036325.2:p.Pro33Ser	missense variant	Benign	1.236%	0.89%	rs61735304	RCV000387944.3
4	FZD4	NM_012193.4:c.313A>G	NP_036325.2:p.Met105Val	missense variant	Pathogenic	0.0043%	0.0%	rs80358284	RCV000210241.1
5	FZD4	NM_012193.4:c.379C>T	NP_036325.2:p.Arg127Cys	missense variant	Likely benign	0.0046%	0.0%	rs376854255	RCV001111581.1
6 New	FZD4	NM_012193.4:c.416G>C	NP_036325.2:p.Trp139Ser	missense variant	(Uncertain significance)	NA	NA	NA	Not in ClinVar
7 New	FZD4	NM_012193.4:c.481G>C	NP_036325.2:p.Gly161Arg	missense variant	(Uncertain significance)	NA	NA	NA	Not in ClinVar
8	FZD4	NM_012193.4:c.502C>T	NP_036325.2:p.Pro168Ser	missense variant	Benign	1.4077%	0.01379%	rs61735303	RCV000368489.2
9 New	FZD4	NM_012193.4:c.1221_1223delTCG	NP_036325.2:p.Ala408del	in-frame deletion	(Uncertain significance)	NA	NA	NA	Not in ClinVar
10 New	FZD4	NM_012193.4:c.1350T>A	NP_036325.2:p.Cys450Ter	stop-gained	(Likely pathogenic)	NA	NA	NA	Not in ClinVar
11	KIF11	NM_004523.4:c.386A>C	NP_004514.2:p.Glu129Ala	missense variant	(Uncertain significance)	0.00004	NA	rs779558239	Not in ClinVar
12	KIF11	NM_004523.4:c.1924C>G	NP_004514.2:p.Pro642Ala	missense variant	Benign	0.02930%	0.0%	rs79865214	RCV000915477.2
13	KIF11	NM_004523.4:c.1578C>A	NP_004514.2:p.His526Gln	missense variant	Benign	0.25320%	0.0013%	rs112145870	RCV000967853.2
14	LRP5	NM_002335.4:c.16C>A	NP_002326.2:p.Pro6Thr	missense variant	Benign	0.2932%	0.0035%	rs771718186	RCV000592927.1
15	LRP5	NM_002335.4:c.34_36CTG [4]	NP_002326.2:p.Leu16_Leu20del	deletion	Uncertain significance	0.0006928%	0.0%	rs72555376	Not in ClinVar
16	LRP5	NM_002335.4:c.58_60dupCTG	NP_002326.2:p.Leu20dup	insert duplication	Benign	10.14%	0.57%	rs564221347	VCV000193231.2
17	LRP5	NM_002335.4:c.266A>G	NP_002326.2:p.Gln89Arg	missense variant	Benign	0.83%	NA	rs41494349	RCV000175719.2
18	LRP5	NM_002335.4:c.1999G>A	NP_002326.2:p.Val667Met	missense variant	Likely benign	3.40%	0.092%	rs4988321	RCV000250939.1
19 New	LRP5	NM_002335.4:c.2543C>T	NP_002326.2:p.Pro848Leu	missense variant	NA	NA	NA	NA	Not in ClinVar
20	LRP5	NM_002335.4:c.2555C>T	NP_002326.2:p.Thr852Met	missense variant	NA	0.0013140%	0.0%	rs1398692057	Not in ClinVar
21	LRP5	NM_002335.4:c.2737dup	NP_002326.2:p.Cys913fsTer73	frameshift variant	Pathogenic	0.0004000%	0.0%	rs886043590	RCV000761295.1
22 New	LRP5	NM_002335.4:c.2754dup	NP_002326.2:p.Ala919CysfsTer67	frameshift variant	(Likely pathogenic)	NA	NA	NA	Not in ClinVar
23	LRP5	NM_002335.4:c.3256A>G	NP_002326.2:p.Met1086Val	missense variant	Likely benign	0.20770%	0.0019730%	rs145774832	RCV000592263.1
24	LRP5	NM_002335.4:c.3989C>T	NP_002326.2:p.Ala1330Val	missense variant	Benign	13.40%	1.60%	rs3736228	RCV000242123.2
25	LPR5	NM_002335.4:c.4565C>T	NP_002326.2:p.Pro1522Leu	missense variant	Uncertain significance	0.02910%	0.0%	rs200624778	RCV000724481.4
26	LRP5	NM_002335.4:c.4488+2T>G	intron 21 donor site lost	splice donor	Pathogenic	NA	NA	rs80358322	RCV000006667.3
27	NDP	NM_000266.4:c.125A>G	NP_000257.1:p.His42Arg	missense variant	Pathogenic	0.0005%	NA	rs104894874	RCV000011437.5
28	ZNF408	NM_024741.3:c.199C>G	NP_079017.1:p.Leu67Val	missense variant	Benign	0.3680%	0.0019710%	rs35652367	RCV000969067.2
29	ZNF408	NM_024741.3:c.581_592del	NP_079017.1:p.Val194_Val197del	deletion	Benign	13.1%	1.59%	rs148055528	RCV000248955.1
30	ZNF408	NM_024741.3:c.689A>G	NP_079017.1:p.Glu230Gly	missense variant	Likely benign	0.0371%	0.0006570%	rs147850078	RCV000974525.2
31	ZNF408	NM_024741.3:c.1010G>C	NP_079017.1:p.Arg337Pro	missense variant	Benign	0.8609%	0.0151100%	rs36017347	RCV000959911.2
32	ZNF408	NM_024741.3:c.1114G>A	NP_079017.1:p.Ala372Thr	missense variant	Uncertain significance	0.0577%	0.0%	rs141624151	RCV001053827.1
33 New	ZNF408	NM_024741.3:c.1940_1941delinsAA	NP_079017.1:p.Pro647Gln	missense variant	(Uncertain significance)	NA	NA	NA	Not in ClinVar

**Table 5:** Single and multigenic variants combinations. Protein-altering variants were found in multiple combinations in many patients. Multiple variants in single genes are indicated within parentheses. Totals are for the numbers of unique, different combinations detected. Genes are listed for the digenic and trigenic variant combinations.

Variant Nature	Gene/s	Total
monogenic	*NDP*	1
monogenic	*LRP5*	16
monogenic	*LRP5* (2)	7
monogenic	*LRP5* (3)	1
monogenic	*FZD4*	2
monogenic	*FZD4* (2)	1
monogenic	*CTNNB1*	1
monogenic	*ZNF408*	1
monogenic	*ZNF408* (3)	1
monogenic	*KIFll*	1
digenic	*LRP5*, *ZNF408*	14
digenic	*LRP5*, *FZD4*	6
digenic	*LRP5*, *KIF11*	2
digenic	*KIF11*, *ZNF408*	1
digenic	*FZD4, ZNF408*	1
trigenic	*LRP5*, *ZNF408*, *FZD4*	2

## Data Availability

For more detalied information on the targeted DNA-sequenicng methodology and process used for this study, as well as the integration of this sequencing service into biomedical science and medical student education, please contact Dr. Kenneth P. Mitton (mitton@oakland.edu).
